# Complete Genome Sequence of a New Ebola Virus Strain Isolated during the 2017 Likati Outbreak in the Democratic Republic of the Congo

**DOI:** 10.1128/MRA.00360-19

**Published:** 2019-05-16

**Authors:** Tony Wawina-Bokalanga, Bert Vanmechelen, Joan Martí-Carreras, Valentijn Vergote, Kurt Vermeire, Jean-Jacques Muyembe-Tamfum, Steve Ahuka-Mundeke, Piet Maes

**Affiliations:** a KU Leuven, Department of Microbiology, Immunology and Transplantation, Clinical and Epidemiological Virology Division, Rega Institute, Leuven, Belgium; b KU Leuven, Department of Microbiology, Immunology and Transplantation, Laboratory of Virology and Chemotherapy, Rega Institute, Leuven, Belgium; c Institut National de Recherche Biomédicale (INRB), Kinshasa, Democratic Republic of the Congo; DOE Joint Genome Institute

## Abstract

Genomic sequencing for early identification of Ebola virus remains a big challenge in low-income countries. Here, we report the complete genome sequence of an Ebola virus strain obtained during the 2017 Likati outbreak in the Democratic Republic of the Congo (DRC) by using the Oxford Nanopore Technologies (ONT) MinION sequencer.

## ANNOUNCEMENT

Ebola virus is a filamentous, enveloped, nonsegmented negative-sense RNA virus that belongs to the genus *Ebolavirus*, family *Filoviridae*. This genus consists of five species, *Zaire ebolavirus* (EBOV), *Sudan ebolavirus* (SUDV), *Bundibugyo ebolavirus* (BDBV), *Taï Forest ebolavirus* (TAFV), and *Reston ebolavirus* (RESTV) (1).

Our research team was deployed to the Democratic Republic of the Congo (DRC) in May 2018 to perform real-time sequencing using the Oxford Nanopore Technologies (ONT) MinION sequencer, supporting local diagnostic teams during the Ebola outbreak. Prior to using the MinION sequencer in Equateur Province, where the outbreak was ongoing, the protocol was validated by sequencing a confirmed Ebola-positive blood sample from the May 2017 Likati outbreak at the Institut National de Recherche Biomédicale (INRB), Kinshasa, Democratic Republic of the Congo.

Of the eight reported cases in the 2017 Likati outbreak, four were fatal, but only two samples have been confirmed to be positive for EBOV (https://www.cdc.gov/vhf/ebola/history/chronology.html). Here, we present the complete genome sequence of the EBOV strain found in one of these two samples, obtained using the MinION sequencer. The other positive sample was deemed inadequate for sequencing due to its low viral load and suboptimal storage conditions.

Viral RNA was extracted from 50 μl of inactivated whole blood using a viral RNA extraction kit (Qiagen) according to the manufacturer’s instructions but with an extra washing step (buffer AW2). Reverse transcription-PCR (RT-PCR) was performed with each of the EBOV-specific primer pools kindly provided by the ARTIC project (2) to amplify the EBOV genome. Pooled amplicons were cleaned up with AMPure XP beads (New England Biolabs) by washing 2 times with 70% ethanol and resuspending in 50 μl of RNase-free water. The purified DNA was quantified on a Qubit 1.0 fluorimeter (Thermo Fisher Scientific), and libraries were prepared using the 1D genomic DNA by ligation (SQK-LSK108) kit and the protocol supplied by ONT. Sequencing was performed using a R9.4.1 flow cell and MinKNOW v2.0 software.

Reads were base called with ONT Albacore v3.0.1 and subsequently quality, adapter, and primer trimmed with Porechop v0.2.3 using default parameters (3). To construct the consensus sequence, *de novo* assembly was performed using Canu v1.7.0 (4), and reference assembly was done with Minimap2 (5) using default parameters. The resulting contigs consist of 896,252 reads with an average depth coverage of 19,604× and a total length of 485,957,350 bp. Reads were mapped against both draft assemblies and posteriorly polished with Nanopolish v0.9.2 (6). BLAST was used to assess the completeness of the draft consensus sequence using the previous EBOV strains (7).

This complete genome is 18,898 nucleotides in length with a GC content of 41.12%, and it consists of a 3′ leader sequence, seven genes (encoding nucleoprotein, viral proteins VP24, VP30, VP35, and VP40, glycoprotein, and polymerase), and the 5′ trailer sequence.

**FIG 1:**
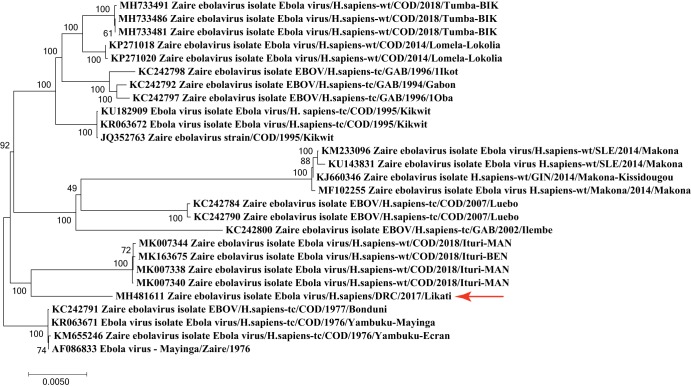
Phylogenetic maximum-likelihood tree based on the Hasegawa-Kishino-Yano model, using a bootstrap value of 500, of the 2017 Likati EBOV sequence (red arrow) compared to that of other full-length EBOV genomes from different Ebola virus outbreaks.

A phylogenetic tree (Fig. 1) was constructed using a multiple-sequence alignment of full-length EBOV genomes by MUSCLE in MEGA v7.0.21 (8), using default parameters. There is a nucleotide similarity of 98.69% between the newly reported sequence and the most closely related genome, EBOV/COD/1976/Yambuku-Mayinga (GenBank accession number KR063671).

### Data availability.

The complete genome sequence of the 2017 Likati EBOV strain has been deposited in GenBank under the accession number MH481611. Raw data were submitted to the National Center for Biotechnology Information Sequence Read Archive (SRA) under the accession number SRR8959866.
